# Efficacy of postoperative pain management in head and neck cancer patients

**DOI:** 10.1186/s40463-018-0274-y

**Published:** 2018-05-02

**Authors:** Ashley Hinther, Steven C. Nakoneshny, Shamir P. Chandarana, T. Wayne Matthews, Joseph C. Dort

**Affiliations:** 1 Department of Surgery, Section of Otolaryngology- Head and Neck Surgery, Cumming School of Medicine, University of Calgary, HRIC 2A02, 3280 Hospital Dr NW, Calgary, AB T2N 4Z6 Canada; 2 Ohlson Research Initiative, Arnie Charbonneau Cancer Institute, Cumming School of Medicine, University of Calgary, Calgary, AB Canada

**Keywords:** Postoperative pain management, Postoperative pain control, ERAS, Enhanced recovery after surgery, Head and neck cancer, quality improvement

## Abstract

**Background:**

Our study quantifies the effectiveness of perioperative pain control in a cohort of patients undergoing major head and neck surgery with free flap reconstruction. Our long-term goal is to improve pain control and thereby increase mobility, decrease postoperative complications and decrease hospital stay.

**Methods:**

A retrospective analysis was performed at a tertiary, academic head and neck surgical oncology program in Calgary, Alberta, Canada from January 1, 2015 – December 31, 2015. Pain scores were recorded prospectively. Primary outcomes were frequency of postoperative pain assessments and pain intensity using the numeric rating scale.

**Results:**

The cohort included 41 patients. Analysis was limited to pain scores recorded from postoperative days 1–14. There was an average of 7.3 pain measurements per day (SD 4.6, range 1–24) with the most frequent monitoring on postoperative days 1–4.

Median pain scores ranged from 0 to 4.5 with the highest median score on postoperative day 6. The daily maximum pain scores recorded ranged from 8 to 10 with scores of 10 recorded on postoperative days 1, 2, 3, 5, 7, 8, and 10.

Patients most frequently had inadequate pain control on postoperative days 1, 2, 4, and 5 with the majority occurring on postoperative day 1.

**Conclusions:**

Postoperative pain control could be improved at our centre. The frequency of pain assessments is also highly variable. Ongoing measurement, audit, and feedback of analgesic protocol effectiveness is an excellent first step in improving perioperative pain management in patients undergoing major head and neck cancer surgery with free flap reconstruction.

## Background

Adequate pain control is a key element in successful recovery after major head and neck surgery. Inadequate postoperative pain management has been correlated with poor functional recovery [1]. Furthermore, continuous unrelieved post-operative pain can activate the pituitary-adrenal axis leading to immunosuppression resulting in postsurgical wound infection and poor wound healing [2–4]. Inadequate pain control can also reduce patient mobility, which can lead to deep vein thrombosis, pulmonary embolism, and pneumonia [5, 6]. Effective postoperative pain control can shorten hospital stay, improve short-term post-operative outcomes, and decrease morbidity [7]. Additionally, poorly managed acute postoperative pain is often associated with chronic pain [8]. Major head and neck cancer resections with free flap reconstruction are lengthy and complex procedures and patients often require nasogastric and tracheotomy tubes. These interventions have a major impact on postoperative patient comfort and can make pain management challenging.

Adequate pain control implies consistent assessment of pain status and reliable delivery of appropriate analgesic medication. Important components of the pain assessment include determining the location of the pain as well as any aggravating or alleviating factors. SeIf-reported pain intensity is the most commonly assessed bedside pain dimension. Anderson et al. found that lack of pain assessment was a major barrier to achieving adequate pain control [9]. Optimal pain assessment requires standardization of schedule and format. Prior authors determined that greater than two pain assessments per day across 4 days is required to have an overall accurate assessment of patients’ pain [10]. Ideally pain is reassessed after each intervention to not only determine the effectiveness of that intervention but also help determine what, if any, additional modifications are needed. Numerous pain intensity measures have been developed and validated. The numeric rating scale (NRS) uses a 0–10 scale to rate the intensity of pain with 10 being the most intense pain [10–13]. As defined by the WHO, poorly controlled pain, or breakthrough pain, is defined as any score on the NRS greater than 3 [14–16].

Pain is prevalent in over 50% of cancer patients with the highest prevalence in patients with head and neck cancer (70%) [17]. Orgill et al. reported that only 35% of post-laryngectomy patients received adequate and effective pain management [18]. Few studies have investigated the effectiveness of pain control in head and neck cancer patients. In most head and neck centers, narcotic analgesics form a major component of postoperative pain control regimens [19, 20]. In our center, similar to others, most patients are managed with intravenous patient controlled analgesia (PCA) for the first five postoperative days and subsequently switched to a combination of narcotic and non-narcotic analgesics (acetaminophen and / or ibuprofen); however, narcotic analgesics have numerous adverse effects that include nausea and vomiting, constipation, sedation, and impaired mobilization [21, 22]. Furthermore, overuse of narcotics in the perioperative period can lead to subsequent drug dependence and its resulting personal and societal impacts.

The objective of this study was to better understand the effectiveness of our current approach to pain management in patients undergoing major head and neck surgery with free flap reconstruction. The type and effectiveness of our drug regimes, and the consistency and reliability of pain evaluation were of particular interest. We hypothesized there would be considerable variability in the evaluation and effectiveness of our approach to pain management. We also believed there would be generalizable findings that would inform our, and others’, practice of pain management in this complex patient population. This information is a critical first step toward improving the overall management of pain in this high-risk patient population.

## Methods

We performed a retrospective study of all patients undergoing head and neck cancer surgery with free flap reconstruction at the Foothills Medical Centre in Calgary, Alberta, Canada from January 1, 2015 – December 31, 2015. Pain assessment scores were collected from an in-hospital electronic medical record for the duration of inpatient stay. Patient demographics and treatment data were collected from a prospectively annotated head and neck cancer database. Primary outcomes were frequency of postoperative pain assessments and pain intensity using the NRS. Secondary outcomes were time to mobilization and length of hospital stay.

Categorical variables are reported as proportions and continuous data are presented with means +/− standard deviation as appropriate. All data were analyzed using Stata version 15 (Stata Corp, College Station, Tx, USA).

The authors used A pRoject Ethics Community Consensus Initiative (ARECCI) framework to assess for and mitigate ethical risks, including the ARECCI Ethics Screening Tool and the ARECCI Ethics Guidelines. The project was deemed a quality improvement initiative with a minimal risk (ARECCI score = 1).

## Results

Clinical characteristics of the cohort (*n* = 41) are found in Table 1. The mean age was 61.2 years with a range of 23–82 years. Pain scores, using the NRS, were analyzed from postoperative days (POD) 1–14.

**Table 1:** Patient demographics and clinical characteristics

Characteristic	Number of subjects (%)
Gender	
Male	32 (78%)
Female	9 (22%)
Age (yrs)	
Mean (SD)	61.2 (12.3)
Range	23.6–82.0
Primary site	
Oral Cavity	23 (56%)
Oropharynx	3 (7%)
Larynx	4 (10%)
Paranasal Sinus	3 (7%)
Skin	3 (7%)
Salivary Gland	2 (5%)
Other Site	3 (7%)
pT Classification	
T0	3 (7%)
T1	6 (15%)
T2	13 (32%)
T3	3 (7%)
T4	11 (27%)
Tx	5 (12%)
pN Classification	
N0	23 (56%)
N1	4 (10%)
N2	7 (17%)
Nx	7 (17%)
Clinical Stage	
0	3 (7%)
I	5 (12%)
II	8 (20%)
III	7 (17%)
IV	13 (32%)
Not Stated	5 (12%)
Length of Stay (d)	
Mean (SD)	11.6 (5.5)
Range	4.0–29.0

The mean length of hospital stay was 11.6 days with a range of 4–29 days. By POD 2, 71% (*n* = 29) of patients were mobilized and 95% (*n* = 39) were mobilized by POD 5.

**Fig. 1:**
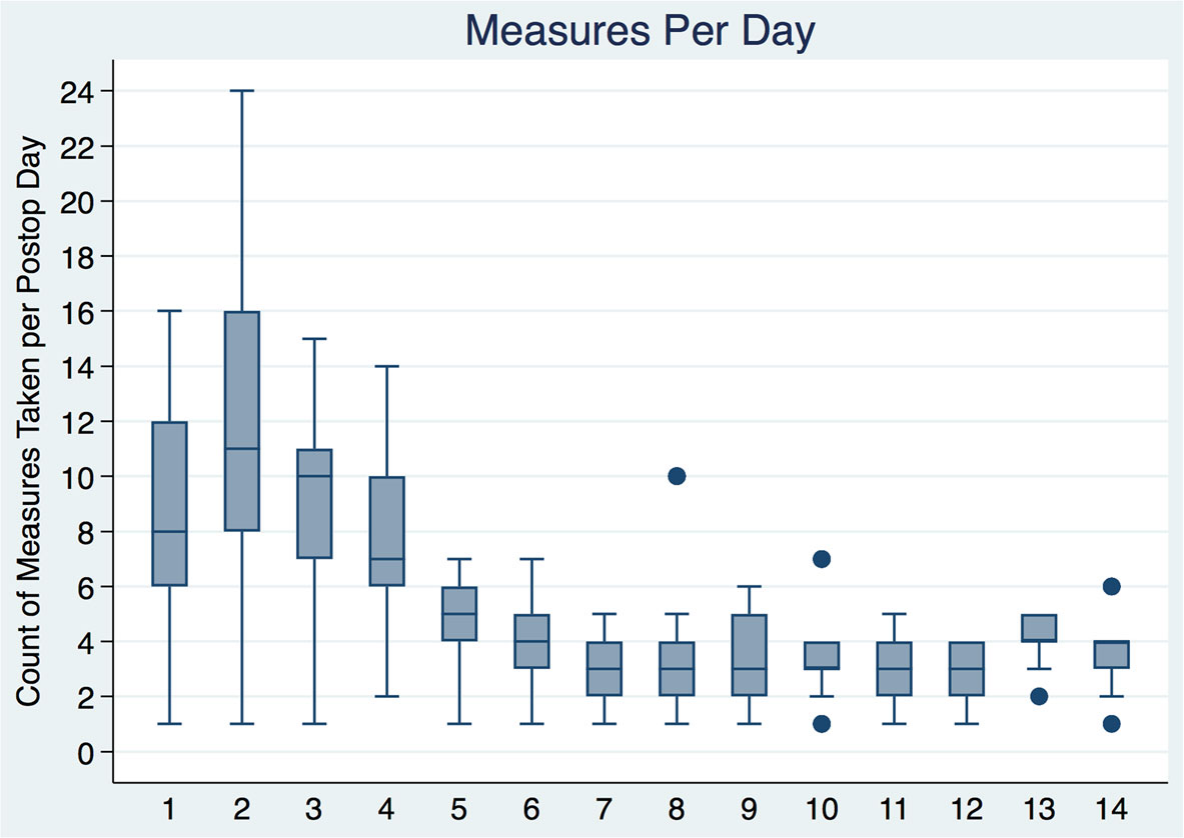
Total number of pain assessments performed per postoperative day

There was substantial variability in the number of daily pain assessments in the postoperative period (Fig. 1). On average, 7.3 pain measurements were performed daily (SD 4.6, range 1–24) with the most frequent monitoring taking place on PODs 1–4.

**Fig. 2:**
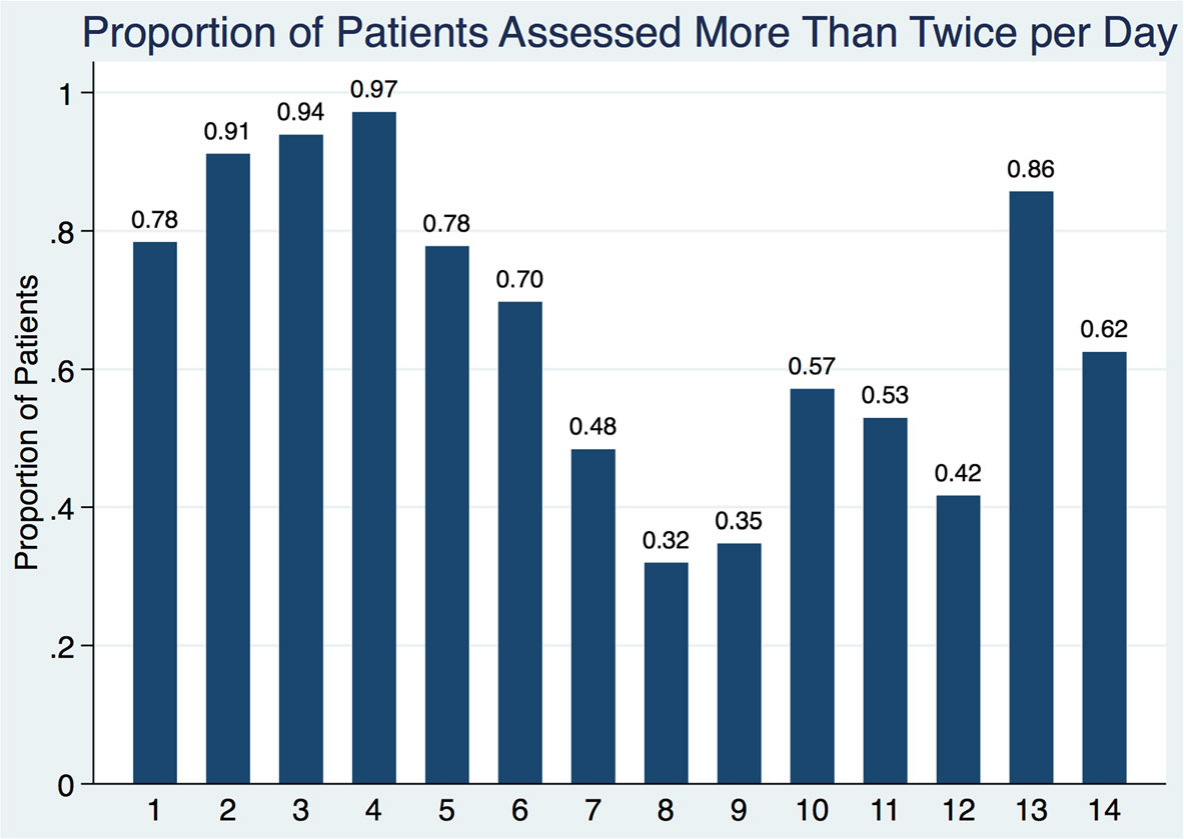
Proportion of patients receiving > 2 pain assessments per postoperative day

Figure 2 illustrates the proportion of patients receiving more than two pain assessments per day. Again, we found large variability in the number of pain assessments, with the greatest proportion of patients receiving more than two assessments per day taking place on PODs 2, 3, 4, and 13. PODs 8 and 9 had the lowest proportion of patients receiving appropriate pain assessments with 32% and 35% of patients receiving greater than two pain assessments that day, respectively. At no time did all of the patients receive an adequate number of daily pain assessments.

**Fig. 3:**
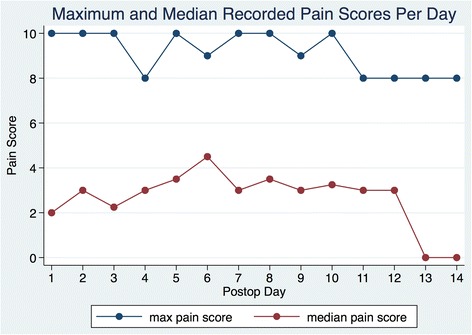
Maximum and median pain scores per postoperative day

Figure 3 shows the maximum and median daily pain scores for all patients. Median pain scores ranged from 0 to 4.5 with the highest median score on POD 6. The daily maximum pain scores recorded ranged from 8 to 10 with scores of 10 recorded on PODs 1, 2, 3, 5, 7, 8, and 10.

**Fig. 4:**
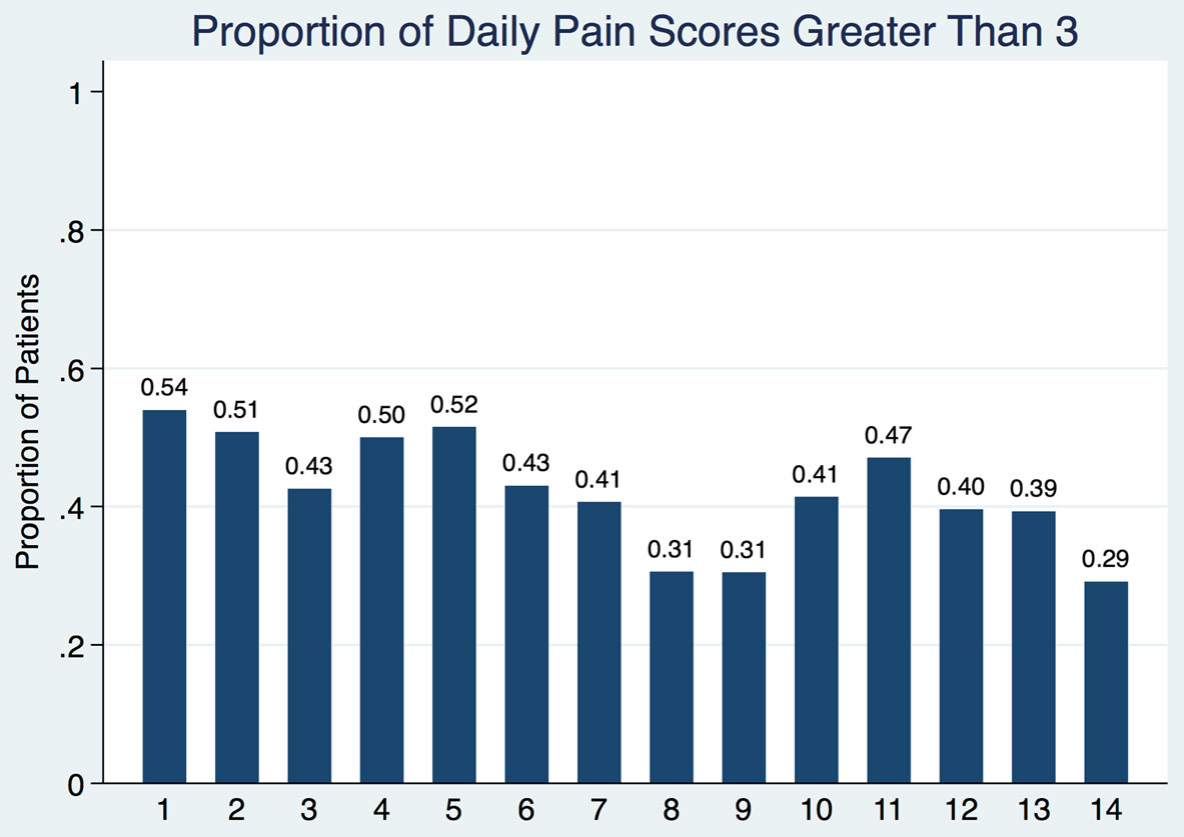
Proportion of mean daily pain scores > 3

Figure 4 demonstrates the efficacy of pain control by indicating the proportion of daily pain scores greater than 3, reflecting poorly controlled pain. 31.5% (531/1684) of the total recorded pain scores were 5 or greater (not shown), signifying moderate to severe pain for at least part of the postoperative period. High scores were observed in 35 of 41 patients, indicating this is a common problem. Poor pain control was most frequent on PODs 1, 2, 4, 5, and 11 with the highest proportion occurring on POD 1. The highest proportion of patients with adequate pain control occurred on POD 8, 9, and 14.

## Discussion

In this study, we found considerable variation in the frequency of pain assessments and the efficacy of pain control. Despite the frequent use of narcotic-based PCA regimes, many of our patients had pain scores greater than 3 with 35 of 41 patients having scores greater than or equal to 5 for at least some part of their hospital stay. These results indicate that current pain management is inconsistent and often ineffective. This study demonstrates there is an opportunity to standardize both postoperative pain assessments and pain management.

Inadequate pain control is a major barrier to a patient’s postoperative recovery and can be a factor in the development of postoperative complications. Appropriate pain control not only takes into account the type of analgesic employed, but also the adequacy of pain assessment in order to ensure the patient’s pain is controlled. Previous studies suggest that despite the existence of guidelines for managing oncologic pain, pain is inadequately treated in nearly half of cancer patients. Head and neck cancer patients have the highest pain prevalence at 70% [14, 23]. These studies outline the importance of critically analyzing current pain management and addressing areas of weakness.

We are currently using the NRS for pain assessment. The NRS is a validated pain assessment tool that is easy to administer and record. Pain scores vary considerably throughout the day; therefore, the NRS must be administered frequently to adequately assess pain control. Jensen et al. demonstrated a minimum of three daily assessments per day should be performed for at least the first 4 postoperative days to provide a reliable pain assessment [24]. Although our results demonstrate a variable number of assessments throughout the day, we also determined there were no significant differences in average pain scores regardless if there were greater than two pain assessments per day (data not shown). We also found there is important inter-patient variability in the number of pain scores recorded per day and considerable intra-patient variability between the numbers of daily pain assessments. The WHO guidelines suggest poorly managed pain is any pain score on the NRS greater than 3 and scores of 5 or greater indicate moderate to severe pain. Figure 4 shows that a meaningful proportion of our patients are spending time in pain states of 3 or greater and our finding that 31.5% of total recorded pain scores were 5 or greater highlights that many patients (35 of 41) likely had less than optimal pain control.

Multimodal analgesic approaches used in other surgical populations minimize the use of narcotics and provide stable, reliable pain control, reduce postoperative nausea and vomiting, and improve ambulation for most patients [24–26]. The complex nature of head and neck cancer surgery suggests pain could be managed through a multimodal analgesic approach [20]. A 2014 randomized controlled study demonstrated decreased opioid requirements and length of hospital stay associated with pre-emptive intravenous paracetamol at the time of induction [27]. The French Oto-Rhino-Laryngology- Head and Neck Surgery Society published guidelines pertaining to the management of postoperative pain in head and neck cancer patients. The French guidelines recommend multimodal analgesia; however, this recommendation is not evidence-based and relies upon professional consensus alone [23, 25, 28]. We therefore believe multimodal analgesia protocols represent an important avenue for further research in the head and neck patient population.

A major challenge in implementing multimodal analgesia is patients’ medical comorbidities, which may contraindicate the use of multimodal protocols. Specifically, any patient with past history of peptic ulcer disease, renal failure, or liver disease will limit the use of NSAIDs and paracetamol.

This study is limited by its retrospective design that prevented an assessment of narcotic-induced complications and side effects. The retrospective design also meant we could not control the nature and frequency of analgesic administration. However, our primary goal was to assess adequacy of pain management and it was apparent that our current approach needs improvement.

This study is strengthened by its use of high quality administrative data that was collected prospectively at the point of care. Such data are highly reliable and we are confident in its accuracy and reliability. Few studies of perioperative pain management in major head and neck cancer surgery with free flap reconstruction have been published. Studies that have been published show a high reliance on narcotic based pain control [8, 9]. We believe that multimodal analgesic protocols, as shown in other surgical disciplines, will reduce the need for postoperative narcotics in head and neck cancer patients and the complications that attend their use [16–18].

## Conclusions

We conclude that in a tertiary academic head and neck surgical oncology program there is significant variation in the number of pain assessments and in the adequacy of pain control in patients undergoing major head and neck cancer surgery with free flap reconstruction. Our results suggest that ongoing measurement, audit, and feedback of analgesic protocol effectiveness is an excellent first step in improving perioperative pain management in patients undergoing major head and neck cancer surgery with free flap reconstruction.

## Abbreviations

ARECCI: A pRoject Ethics Community Consensus Initiative
NRS: Numeric rating scale
PCA: Patient-controlled analgesia
POD: Postoperative day
WHO: World Health Organization
